# Biomimetic Phantoms in X-Ray-Based Radiotherapy Research: A Narrative Review

**DOI:** 10.3390/biomimetics10120794

**Published:** 2025-11-21

**Authors:** Elisabeth Schültke

**Affiliations:** Department of Radiooncology, Rostock University Medical Center, 18059 Rostock, Germany; elisabeth.schueltke@med.uni-rostock.de

**Keywords:** anatomic mimicry, biomechanical mimicry, biomimetic principles, dosimetry, phantom, quality assurance (QA), radiotherapy, tumour environment

## Abstract

The field of experimental radiooncology and the quality assessment (QA) aimed at patient safety both profit from the utilisation of biomimetic principles. The work with phantoms based on biological structures of animals or humans, utilising the principles of anatomic mimicry, has a long tradition in radiotherapy research. When phantoms are produced from tissue-equivalent materials, they mimic the radiological properties of tissues and organs, allowing researchers and clinicians to study dose distribution and optimise treatment plans without exposing real patients to radiation. Biomechanical mimicry would take this a step further by creating phantoms that replicate the movement and deformation of organs during physiological movement, such as heartbeat or breathing, enabling a more accurate simulation of dynamic treatment scenarios. Bioinspired sensor technologies, such as artificial skin or integrated detectors, can be used to monitor radiation exposure, organ motion or temperature changes during therapy with high precision. The utility of such a phantom could be further enhanced by creating a realistic tumour microenvironment as an irradiation target, following the principles of microenvironmental biomimicry. Thus, biomimetic strategies can be exploited in the validation of radiotherapy technologies and open new perspectives for adaptive radiotherapy and real-time monitoring.

## 1. Introduction

In clinical radiotherapy, phantoms are well-established tools for dose measurement (dosimetry) and quality assurance (QA), often during the commissioning process for new equipment, but also to maintain patient safety in an ongoing process through repeated measurements. They are used to verify the accuracy of dose delivery, calibrate imaging and therapy systems and model dose distribution. In addition, phantoms are valuable educational tools. If these phantoms simulate the properties of human or animal tissue, they are said to be biomimetic phantoms. A distinction is made between tangible phantoms existing as three-dimensional structures and virtual phantoms.

Virtual phantoms have some advantages over physical phantoms. In physical phantoms based on patient data, the resemblance to the ‘data donor’ might allow for identification, which is much less likely with virtual phantoms. Once created, they may exist unaltered for long periods of time. Alternatively, they can be updated based on new data sets, with the aim to improve the extent of biomimicry. In either case, they are very likely to deliver reproduceable results. Virtual phantoms can be used in training situations to simulate different real-life scenarios without endangering a patient if the trainee makes a wrong decision. Clinically relevant scenarios like tumour growth and organ shift can easily be simulated once a sufficient amount of real-life data has been introduced. Such highly accurate modelling comes at a high computational cost, especially where Monte Carlo simulation is involved. The modelling process might require significantly more time than creating a physical model of an individual patient. In the setting of personalised medicine, the physical model might be the better choice, reproducing detailed anatomic information in a time-efficient manner.

The requirements for phantoms used in research and those used in daily QA routines can differ widely. The specifications of phantoms used in daily radiotherapy QA routines, for instance, are guided by AAPM Task Group reports [1,2] and IAEA guidelines [3]. They typically adhere to the national standards. Phantoms created for specific research and development purposes, on the other hand, do not necessarily adhere to any clinical standards and there is no need to certify the tool prior to use. A common denominator for both types of phantoms is data reproducibility.

The focus of this article is on biomimetic phantoms that are specifically developed or modified to improve the understanding of processes driving new, still experimental, X-ray-based radiotherapy techniques. Mimicry as a key to phantom development is discussed in examples of both physical and virtual phantoms.

One could certainly argue whether virtual phantoms can be considered biomimetic. What evidence do we find when looking at the different types of virtual phantoms?

## 2. Virtual Phantoms in Radiotherapy Research

Virtual phantoms used in radiotherapy research are computer-generated models simulating the anatomy and/or physical properties of human or animal tissue for use in the planning and quality assurance of clinical radiotherapy, as well as in experimental radiotherapy. Unlike physical, tangible phantoms, virtual phantoms are software-based and exist in virtual space only. They can be used to simulate radiotherapy-associated processes like dose distribution and tumour development in response to irradiation. Thus, they may be used to evaluate and benchmark dose calculation algorithms in treatment planning systems (TPS) or Monte Carlo codes.

A PubMed search conducted for the keywords ‘virtual phantom’, ‘radiotherapy’, ‘research’ and ‘X-ray’ provides an overview of the different types of virtual phantoms, including voxel-based phantoms, mathematical phantoms and hybrids of these two types. Anthropomorphic phantoms and virtual phantoms reproducing the specific anatomy of veterinary patients may be grouped separately.

A PubMed search for ‘voxel-based phantoms’, ‘radiotherapy research’ and ‘X-rays’ alone returned 160 publications; the first one was recorded in 1996 [4]. The validation of a first high-resolution voxel-based head phantom was published in 2000 [5]. In voxel-based phantoms, a human or animal body is modelled as a 3D grid of volume elements (voxels), where a specific density can be assigned to each voxel [6]. These values can be standardised in a ‘one-fits-all’ manner or be patient-specific. In either case, they are based on anatomic data and transferred into virtual space. Spatial resolution is determined by voxel size. Voxel-based phantoms feed on data obtained in imaging procedures, like computed tomography (CT), magnetic resonance imaging (MRI) and positron emission tomography combined with computed tomography (PET-CT). Voxel-based phantoms have been used to assess the impact of tissue types and their individual distribution on dosimetry [7], out-of-field dose measurements in a paediatric patient population [8] and to assess the X-ray exposure of patients during cone beam CT, an imaging procedure used for fast patient position verification preceding the actual irradiation procedure, often as a daily occurrence [9]. This is possible because voxel-based phantoms provide high anatomical fidelity. Furthermore, they are compatible with DICOM-based radiotherapy. They require considerably large computer space though, for data storage and analysis. For reference dosimetry, a much simpler generic mathematical phantom would suffice.

Mathematical phantoms use equations and geometric shapes like spheres and cylinders to represent organs and are less anatomically accurate. An early example was created by Snyder and colleagues [10]. But how much does the accuracy of the phantom weigh in as a decisive criterion for a phantom to be considered biomimetic? After all, a sphere is an abstraction of a head, just without some of the individual external features giving it a distinctive personal touch. This question could perhaps be answered using a third group of phantoms, called hybrid phantoms. They combine the elements of voxel-based and mathematical phantoms. This results in deformable anatomical models, permitting the simulation of realistic scenarios to be studied, including the effects of breathing, organ filling, or patient movement. The availability of such phantoms can greatly aid in time-resolved (4D) radiotherapy planning and adaptive radiotherapy, for conventional clinical radiotherapy and novel radiotherapy techniques [11,12,13]. During training sessions, adaptive strategies can be developed by simulating anatomic changes over time [14]. Considering recent advances in the field of machine learning, it is not only the humans learning. Deep learning frameworks are already able to support real-time image-guided radiotherapy (IGRT) by predicting and anticipating motion [15] and they allow for the real-time correlation of the surface motion and inner organ deformation [16]. For the purpose of working with deformable phantoms, the smaller computer footprint of mathematical phantoms is clearly an advantage over voxel-based phantoms.

If patients are admitted for re-irradiation in the setting of tumour recurrence, hybrid phantoms can aid in dose reconstruction [17], which would be especially useful where patients have been previously irradiated on different irradiation systems or in different radiotherapy centres. Even though these phantoms function exclusively in virtual space, the data they are based upon are real-life data.

Last but not least, there are anthropomorphic (human shaped) virtual phantoms, designed to mimic realistic human anatomy across populations. While the primary purpose of such phantoms might be education and training, they have also been used in multicentre studies and population-based dosimetry. To support the latter, age- and population-specific phantoms have been created [18,19]. Research themes include an improved understanding of the impact on patients caused by radiation exposure during CT scans associated with radiotherapy [20], estimating the secondary cancer risk associated with radiotherapy [20] and understanding the late effects of radiotherapy [21]. All these phantoms are based on human or animal data. What they do not include is tangible physical mimicry [22]. However, modern design software and 3D printing techniques can help to convert virtual anatomic phantoms, based on anonymised patient data, into physical phantoms [23]. These phantoms may act as a twin, capable of reproducing the physical irradiation environment of the original. Twin phantoms may be used in the comparison of treatment plans with a different dose distribution, without the need to use live animals. They could greatly contribute to replacement and reduction in animal experiments. In intraoperative radiotherapy, twin phantoms may be used as training tools and to increase patient safety [24].

## 3. Physical Phantoms and Mimicry

Not all physical phantoms are twins. Physical radiotherapy phantoms may be very simple, representing only one or two important key parameters, depending on the requirements of the study or the QA process it is meant to serve. Some physical radiotherapy phantoms, like the water phantom that is typically used in the daily QA procedure in clinical radiotherapy, may not look at all like a real-life irradiation target (a human or animal body). Those water phantoms or solid water equivalents are widely available and easy to set up. Based on their uniform density, they offer excellent data reproducibility. The dosimetric characteristics are well-understood and the International Commission on Radiation Units and Measurements (ICRU) recommends water as the reference medium for dosimetry standards [25]. However, they lack patient-specific morphology. Facial features and their underlying bone structures, for instance, are part of this individual morphology. They contribute significantly to beam attenuation. As a result, simple phantoms may lead to overestimation of the delivered dose.

More complex phantoms, on the other hand, are often based on the mimicry of one or more key components of the real biological irradiation target, thereby following biomimetic principles. Designed to simulate study-specific key parameters determining the interaction of radiation with tissue, like tissue density, beam attenuation and beam scattering, these phantoms are useful during the validation process of more complex radiotherapy plans, especially in the assessment of new radiotherapy techniques and in the setting of personalised medicine.

Radiotherapy planning and the actual irradiation process are typically preceded by a series of imaging procedures, such as simple X-ray radiography, CT or MRI scans and, in some cases, even PET-CT. If a radiotherapy phantom is used in diagnostic imaging modalities, in an end-to-end testing process, it becomes essential that the materials used are compatible with those imaging modalities with respect to radiological contrast, spatial resolution and dose attenuation [26,27,28,29,30]. Where the phantom is not only used for technical dose measurements, but for the correlation of the measured dose and radiobiological effects, a simulation of both anatomic key features and the biological tumour environment may be helpful [31,32].

### 3.1. Anatomic Mimicry

If the shape of a phantom mirrors the anatomic-morphologic structure, shape and overall size of the biological irradiation target, this is called anatomic mimicry. If the phantom is inspired by an irradiation target in the human body, it is called anthropomorphic. But why is this important in radiotherapy?

**Figure 1:**
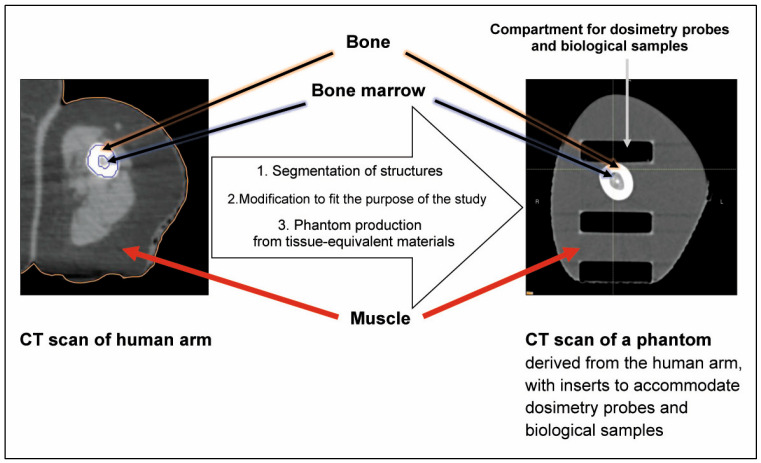
Production of a human arm phantom as example of anatomic mimicry.

Size, shape and tissue composition determine the extent of dose absorption and are, therefore, key parameters in medical physics radiotherapy planning, which is important for both dose deposition in the target and dose attenuation caused by tissues located between the dose entry point and the irradiation target. Whole body phantoms or phantoms of specific body parts, used for QA processes as well as for research purposes, would be classic examples [33,34,35]. These phantoms are typically produced from tissue-equivalent materials to simulate key characteristics of organic tissue, like electron density and X-ray absorption. A complex study conducted in skin, a seemingly simple organ, highlights the many parameters influencing its response to radiotherapy [36]. In the process of end-to-end QA for new irradiation techniques with complex dose distributions, the optimal phantom would not only match the overall size and external shape of the biological irradiation target, but is also representative of tissue types (bone, soft tissue, fat) and the internal anatomy (including bones, air cavities and fluid-filled spaces). Phantoms designed from tissue-equivalent materials may match the electron density and Hounsfield units (HU) on CT, and sometimes even contrast MRI. The specific requirements for phantoms in imaging and therapy studies have been reviewed [37] and a good overview of Hounsfield units matching commonly used materials to produce tissue equivalents with additive manufacturing (3D printing) techniques can be found in the literature [38,39,40,41,42]. Acrylonitrile butadiene styrene (ABS) and polylactic acid (PLA), for instance, are well-suited to represent brain tissue [38]. Their densities and Hounsfield units (CT numbers) are shown in Table 1. Gypsum (CaSO_4_·2H_2_O), or a mixture of gypsum and silicone, can be used to simulate bone structure [43,44,45]. Alternatively, PLA or reinforced PLA may be used to model bone [46]. As shown in Figure 1, cortical bone was simulated by gypsum (CaSO_4_), while a mixture of Vaseline (75 wt.%) (Carl Roth GmbH + Co. KG, Karlsruhe, Germany) and dipotassium hydrogen phosphate (K_2_HPO_4_) (25 wt.%) can be used to simulate bone marrow [44], consisting of gypsum (CaSO_4_). Some of these materials, like PLA and ABS, are available as printable filaments and can be used to generate phantoms on a 3D printer. Cavities can be included into the design, to be later filled with fluid (to simulate the lateral ventricles in the brain, for instance) or to be filled with gypsum to simulate bones. An alternative is to print with two different types of filaments, with one of these being water-soluble. The latter technique, in which cavities are created only at the end of the printing process by dissolving the water-soluble substance, is of an advantage where thin walls or lamellae are to be produced, because, if the entire phantom is 3D-printed as one solid block, it is much less likely that the thin walls will collapse and ruin the printing project.

All values were obtained at 120 kV.

The physical density of tissue is proportional to its absorption X-ray beams. It determines the percentage of X-ray energy attenuation between skin entry and the irradiation target. Using an energy-dependent transformation coefficient, a Hounsfield unit (HU, CT number) is calculated. The infill percentage of calcium-doped PLA is directly proportional to the HU. CT densities ranging from approximately −227 HU to 851 HU can be produced by modifying a calcium-doped PLA filament. Distilled water has been defined as zero HU. The density of tissue-equivalent materials can be modified by adjusting the infill percentage.

To ensure that the phantom behaves similarly to real tissue when exposed to ionising radiation, materials with similar densities and CT numbers, compared to human tissue, were used to produce this human arm phantom for experimental radiotherapy at the synchrotron. Modular inserts were designed to accommodate either technical dosimetry probes, like microDiamond (60019 PTW, Freiburg, Germany) [50] or X-Tream dosimeter (CMRP Wollongong, Australia) [51] and Eppendorf tubes containing biological samples in the same position. This allowed a good correlation between measured dose values and the radiobiological response. An interesting point was raised in a study comparing the water equivalence of polystyrene, PMMA, and solid water as phantom materials for 192Ir dosimetry in brachytherapy: not only was water equivalence material-dependent, but it was found to also be size-dependent [52]. Furthermore, in the same study, water equivalence seems to depend on the distance from the irradiation source.

Anatomically correct phantoms can greatly enhance the quality of dosimetry in irradiation techniques producing a non-homogeneous dose distribution pattern. This applies to already clinically well-established brachytherapy techniques, as well as to the new spatially fractionated radiotherapy (SFRT) techniques like LATTICE, minibeam and microbeam radiotherapy (MRT). In these cases, the effort to produce a patient-specific phantom seems justified, because anatomic components like bone, fluid–solid interfaces and air cavities would have a significant impact on dose distribution, with respect to beam attenuation and scattering in the tissue. They would modify the dose deposited in the irradiation target as a percentage of the skin entry dose. In the correlation of the measured dose and radiobiology effect, dosimetric accuracy is an important factor. Anatomically twinning the phantom to the original patient, veterinary or human, would improve the dosimetric accuracy. For irradiation in mobile targets, like in lung cancer, a simulation of physiologic movement will significantly improve the dosimetric accuracy.

Dosimetry in lung tissue, for instance, is a veritable challenge in SFRT. ABS has been used to model lung tissue in a 3D-printed thorax phantom containing tissues of different densities, also including soft tissue and bone [49]. This phantom was successfully tested in kV imaging and MV photon dosimetry: a typical setting for radiotherapy planning. Another realistic scenario could be the inclusion of alginate hydrogel foams, which can be modified to mimic lung tissue at different stages of emphysema by varying the air volume ratio [53]. Since the majority of patients with lung cancer have been active smokers for many years, lung emphysema is a frequent comorbidity that is relevant in radiotherapy treatment planning.

The use of hydrogels has also been proposed for other soft tissue structures and organs. Polyvinyl alcohol (PVA) hydrogels, modified by repeated freeze/thaw cycles, have been proposed as a substitute for prostate tissue [54]. Interestingly, other authors reported blood vessel substitutes based on a PVA cryogel [55]. In the field of neuroscience, hydrogel has been used to understand the process of substance distribution in the brain parcenchyma, in the absence of active transport processes [56]. Different hydrogel formulations were tested as a brain tissue surrogate in a brain trauma study, alongside PLA [57].

Data were acquired from an anonymised human patient CT scan, using a Brilliance CT Big Bore Oncology scanner (Philips, Hamburg, Germany), operating at 290 mA and 120 kV. Image segmentation was conducted based on density (CT numbers), using 3D Slicer software (Alliance for Medical Image Computing (NA-MIC), Boston, MA, USA). Reproduced tissue types included bone, bone marrow and soft tissue. The CT scan acquired from the phantom, using the same CT scanner, showed that Hounsfield units in the phantom are close to those in the original CT. The three compartments can accommodate modular inserts that are specifically designed to fit a microDiamond dosimeter (PTW, Freiburg, Germany), radiochromic film or/and biological samples contained in small tubes. The detailed design and production process of this phantom has been described in [44].

Relevance to development and QA: Reproduces key parameters of medical physics treatment planning in radiotherapy—size, shape and electron density of a real human body part. The production process is suited for patient-specific modelling. Limitation: If produced as component of an individualised treatment approach, a considerable amount of waste is produced, because the body part is patient-specific.

### 3.2. Biomechanical Mimicry

**Figure 2:**
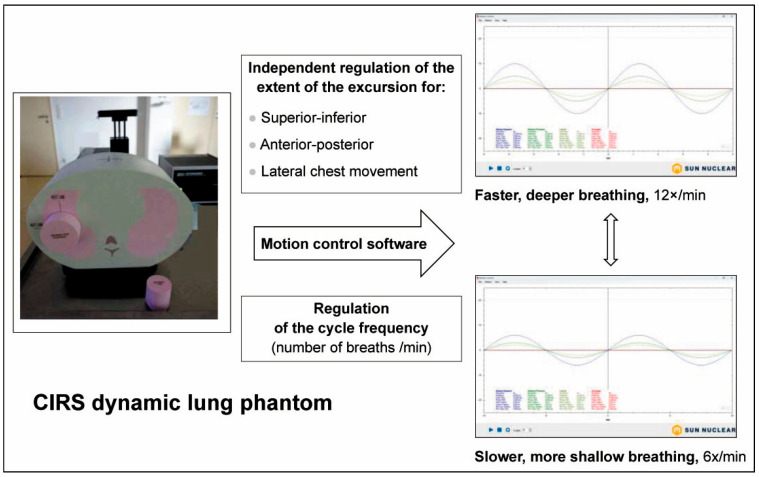
The CIRS dynamic chest phantom as an example of biomechanical mimicry.

One of the challenges in radiotherapy planning is the precise, accurate irradiation of moving targets. Target movement within an irradiation fraction is typically caused by physiologic movements, like heartbeat and breathing. Phantoms reproducing these physiologic movements use biomechanical mimicry. They can be extremely valuable in the validation of new irradiation procedures and techniques involving moving irradiation targets. A typical example for a commercially available biomimetic phantom would be the CIRS dynamic lung: an anthropomorphic thorax phantom composed of tissue-equivalent materials that can accommodate a set of moving tumour target equivalents [58]. This phantom comes with a software packet for the simulation of natural breathing patterns by the modification of anterior–posterior, superior–inferior and lateral thorax wall movement, as well as the duration of breathing cycles (Figure 2). Since the accurate and precise dose delivery to movable targets is a challenge in radiotherapy and lung cancer is the cancer with the most frequent deaths worldwide [59], it is not surprising that the number of dynamic thorax phantoms is relatively high [58,60,61,62], compared to phantoms of the liver [63,64] or the heart [65,66].

Anatomic and biomechanic mimicry in radiotherapy phantoms can aid significantly in validating imaging algorithms, treatment planning systems, and dose delivery accuracy in moving targets.

Spherical targets of different diameters, contained in a small pink capsule like the one seen in front of the chest phantom, can be inserted into the piston-moved cylinder protruding from the phantom. The extent of lateral, anterior–posterior and superior–inferior chest excursion in mm (Y-axis) and the length of each breathing cycle in seconds (X-axis) can be regulated via motion control software (Sun Nuclear). The software examples illustrate different breathing patterns generated by the modification of key parameters like respiratory rate and the extent of thorax wand movement in different directions.

Relevance to development and QA: Allows realistic simulation of the motion range of a mobile target within the chest under different conditions (well-rested, agitated, etc.). Limitation: The target position is defined by the manufacturer. Not suited for patient-specific studies/personalised medicine.

### 3.3. Simulating the Tumour Microenvironment

From a clinical perspective, the radiobiological validation is the most important component of the phantom work, because it ties in directly with the potential success of the treatment. Treatment planning often relies on a simplified assumption of tissue homogeneity. However, in reality, tissues are not at all homogeneous. The efficacy of radiotherapy is significantly modulated by the heterogeneous and dynamic nature of the TME, which includes a variability in tissue oxygenation, cellular composition, extracellular matrix components and vascular structure [67,68]. Factors like hypoxia, stromal interactions and immune response influence the outcome after radiotherapy [69,70]. Therefore, creating phantoms that faithfully mimic the TME is critical for improving radiotherapy dosimetry and treatment outcomes. To better understand and predict TME-specific effects, researchers have developed phantoms replicating the complex architecture and biological properties of the TME. This is called biomimicry. The design and production of TME-mimicking phantoms can facilitate a better understanding of tumour biology, in general, and of radiobiology, in particular. These phantoms may be used to correlate the measured technical dose with the radiobiological response to irradiation.

TME mimicry can serve as a valuable tool in the development of novel therapeutic strategies, and can accelerate the development of personalised therapies. This applies to very basic TME phantoms, with cell cultures presented in a plexi phantom [71], and to rather elaborate phantoms, combining anatomic and even biomechanic mimicry with mimicry of the tumour environment [72,73,74,75].

**Figure 3:**
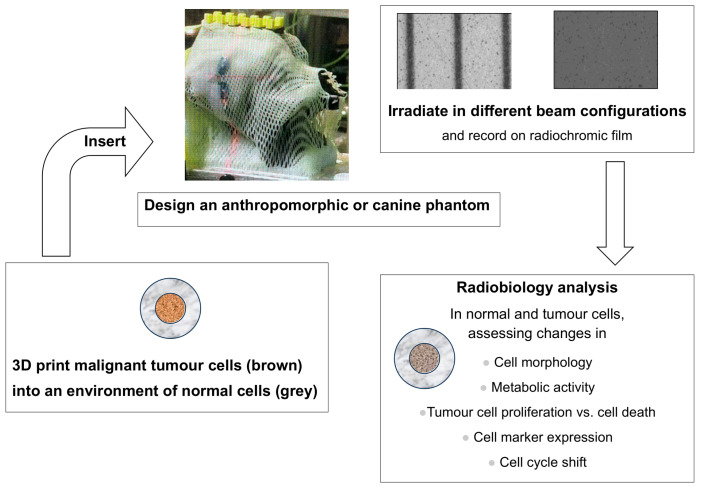
Schematic of phantom development using biomimicry and anatomic mimicry. Design and production of the canine phantom have been described in [77] and the 3D-printed tumour in an environment of normal cells has been described in [31].

The optimal radiotherapy phantom of the future, especially for the use with SFRT concepts, might feature personalised anatomic features, a biomechanical setup and 3D cell cultures grown from the patient’s own tumour cells. In such a setup, even the combined concepts of radiotherapy and systemic therapy (chemotherapy/immunotherapy) could be tested. According to a recent review, more than two thousand publications can be found for 3D printing in cancer research and therapy, including the growing field of 3D bioprinting [76]. An example of using technical and biological 3D printing methods to correlate technical dosimetry, information on beam geometry and radiobiology response in one single phantom was developed by Bustillo and colleagues [77]. The phantom was designed using anatomical mimicry based on a veterinary patient’s diagnostic CT and MRI scans. Three-dimensionally printed from tissue-equivalent materials, it contains modular inserts along the midline to accommodate either microDiamond dosimetry probes, silicon strip detectors or radiochromic film. What makes this phantom special is that the phantom twins the absorption characteristics of the original dog’s head. The phantom can integrate PCR tubes containing biological samples in the same position as the dosimetry probes, allowing for irradiation of the dosimeter and the biological sample under precisely the same conditions. Irradiating tumour cell samples in this phantom, one can see the patient-specific dose distribution patterns. In the future, those inserts could be modified to also include 3D TME samples (Figure 3).

Two already-stablished phantoms, a dog head based on anatomical mimicry and a radiobiology model using biomimicry, presenting cancer cells in a normal cell environment could be combined in future radiobiology studies. Thus, an anatomically correct phantom produced from tissue-equivalent materials, providing key parameters for medical physics therapy planning like real-life size and shape of the patient, can be combined with an interesting radiobiological scenario at a realistic depth-from-surface to obtain valuable data for future veterinary trials. The 3D-printed tumour phantom can be inserted into one of the modular inserts that are visible as rectangular shapes on the top of the head. Currently, they can either accommodate dosimetry probes or biological samples and radiochromic film at the same position. An insert accommodating 3D tumour models, however, could be easily created. The measured dose and radiobiologic response can then be correlated and the irradiation geometry can be recorded on radiochromic film. In our example, the recordings are for microbeam radiotherapy (MRT, left) and broad beam radiotherapy (right). In preparation for irradiation, a clinical thermoplastic mask system can be fitted to the head phantom, to assure reproducible positioning.

Relevance to development and QA: Optimal for radiobiology studies, as it combines the anatomical factors that are relevant in radiotherapy planning (size, depth-from-surface, electron density of tissues/tissue-equivalent materials) with a relevant biological target. Limitation: In the current version, there are no options to model physiological and biochemical feedback mechanisms.

Phantoms combining anatomical, biomechanical and TME mimicry most closely simulate the real-life environment. Data obtained in such systems are most likely to allow for a more realistic assessment and prognosis for success in bench-to-bedside translation than simplified phantoms and should therefore be used wherever possible.

## 4. Which Phantom Serves Best?

Since 2006, more than 2000 phantoms have been reported in cancer research [73]. This reflects the many aspects of cancer research in need of modelling. When using a simple water-based phantom without inhomogeneities, like those caused by different tissue densities, and without interfaces between solid, fluid and air phases, the error margin is relatively small in most parts of the treatment volume [78,79]. Measured in a 30 × 30 × 20 cm solid water phantom and in a water tank at 120 kV, they yield dose deviations within 1–3% when compared to treatment planning system (TPS) predictions under uniform irradiation conditions [80]. Considering the ease of use of these phantoms and their data reproducibility, they are particularly useful in daily QA protocols. However, simple phantoms perform unsatisfactorily in moving targets and complex anatomies, at interfaces of different tissue densities, for narrow beams like minibeams or microbeams and at steep dose gradients [81]. Heterogeneous anthropomorphic, tissue-equivalent phantoms closely mimic the irradiation target in terms of external geometry, internal structure, tissue composition, and imaging characteristics. They incorporate tissue equivalents of different densities like lung, bone and soft tissue analogues, and thus provide a realistic platform for imaging and dosimetry in an end-to-end QA process, although errors can increase substantially at tissue interfaces and within low-density regions, in small fields and in mobile targets [28,82,83]. In a study using anthropomorphic female pelvic phantoms, an average deviation of 6.9% was reported in the heterogenous version, compared to less than 3% in a homogeneous phantom version under identical planning conditions [84]. Even more strikingly, small field irradiation through heterogeneous layers can produce deviations exceeding 25%. When simple calculation algorithms were used [85], up to 40% error was observed in bone-in-water slab configurations under orthovoltage conditions [86]. Motion further amplifies the error risk, but this can be reduced by Monte Carlo-based algorithms [87,88].

Contrary to the ubiquitarian water phantoms, most biomimetic phantoms are not mass-produced but designed to perform in a specific setting of clinical or experimental radiotherapy.

## 5. Conclusions

The development of biomimetic phantoms in radiotherapy is driven by the quest for new irradiation technologies and individualised medicine. The closer a phantom mimics the different components of increasingly complex therapeutic approaches, the higher the likelihood is that the data obtained with this phantom are representative of the real-life situation. In other words, the better the phantom dedicated to the exploration of new irradiation techniques, the more realistic the expectations for the potential outcome in clinical situations are. Virtual phantoms, based on data obtained in physical, tangible phantoms, allow for easy, almost barrier-free transport for interactive anatomical, physiological and biochemical information in the field of radiotherapy. They are perfect tools for therapy plan simulations. The validation of the simulation results, however, needs to be conducted in physical phantoms, delivering tangible data. This process has already been standardised for clinical radiotherapy. For the research and development stage of newly introduced radiotherapy techniques, however, we are free to define the validation process to best suit our need for information. The optimal information gain can be expected from biomimetic phantoms combining anatomical mimicry with simulation of the tumour environment and, where appropriate, with biomechanical mimicry. The choice or the design of a phantom depends on the purpose of the study, the test environment and, obviously, on the availability of the phantom itself, physical or virtual. All those phantoms have one very important aspect in common: their utility in the QA process and thus, eventually, their contribution to improving knowledge, understanding and patient safety.

## Figures and Tables

**Table 1:** Overview showing density values and Hounsfield units (CT numbers) for brain, bone, fat and soft tissue, PLA and ABS.

Organic Tissue	Density [47]	Hounsfield Units [47]	Substitute	Density [48]	Hounsfield Units
**Brain**	1.04	Average 38 White matter 25, Grey matter 40	ABS, 80–100% infill [48] or PLA or PLA calcium doped infill 50–60% [49]		matched by modifying the percentage of infill
**Bone, solid**	1.33–1.68	544–1092	PLA stone PLA chalk PLA 90–100% infill [48]	1.64 1.39 1.24	1063 537 [48]
**Fat**	0.95	−75	ABS, 80–100% infill [48] or PLA or PLA calcium doped infill 50–60% [49]	1.07	30 [48]
**Soft tissue (muscle)**	1.05	43	PLA or PLA calcium doped, infill 50–60% [49]		matched by modifying the percentage of infill
					
**Water**	1.0	0			

## Data Availability

Not applicable.

## Abbreviations

The following abbreviations are used in this manuscript:

AAPM	American Association of Physicists in Medicine
ABS	Acrylonitrile butadiene styrene
CT	Computed tomography
HU	Hounsfield units
ICRU	International Commission on Radiation Units and Measurements
IGRT	Image-guided radiotherapy
MRI	Magnetic resonance imaging
MRT	Microbeam radiotherapy
PET-CT	Positron emission tomography, combined with a CT scan
PVA	Polyvinyl alcohol
QA	Quality assurance
SFRT	Spatially fractionated radiotherapy
TME	Tumour microenvironment
TPS	Treatment planning system

## References

[1] Smith K., Blasi O., Casey D., Chan M., Dieterich S., Dresser S., Kennedy C., Kielar K., Lowenstein J., Pawlicki T. (2025). AAPM Task Group Report 332: Verification of Vendor-Provided Data, Tools, and Test Procedures in Radiotherapy. Med. Phys..

[2] Brock K.K., Mutic S., McNutt T.R., Li H., Kessler M.L. (2017). Use of image registration and fusion algorithms and techniques in radiotherapy: Report of the AAPM Radiation Therapy Committee Task Group No. 132. Med. Phys..

[3] World Health Organization (2021). Technical Specifications of Radiotherapy Equipment for Cancer Treatment.

[4] Kawrakow I., Fippel M., Friedrich K. (1996). 3D electron dose calculation using a voxel based Monte Carlo algorithm (VMC). Med. Phys..

[5] Evans J.F., Blue T.E., Gupta N. (2001). Absorbed dose estimates to structures of the brain and head using a high-resolution voxel-based head phantom. Med. Phys..

[6] Fanti V., Marzeddu R., Massazza G., Randaccio P., Brunetti A., Golosio B. (2005). A simulator for X-ray images. Radiat. Prot. Dosim..

[7] Ferrauche G., Tramontin G., Massera R.T., Tomal A. (2023). Impact of fibroglandular tissue distribution and breast shape in voxelized breast models for dosimetry in mammography. Phys. Med. Biol..

[8] Cravo Sá A., Barateiro A., Bednarz B., Borges C., Pereira J., Baptista M., Pereira M., Zarza-Moreno M., Almeida P., Vaz P. (2020). Assessment of out-of-field doses in radiotherapy treatments of paediatric patients using Monte Carlo methods and measurements. Phys. Med..

[9] Baptista M., Di Maria S., Vieira S., Santos J., Pereira J., Pereira M., Vaz P. (2019). Dosimetric assessment of the exposure of radiotherapy patients due to cone-beam CT procedures. Radiat. Environ. Biophys..

[10] Snyder W.S., Fisher H.L., Ford M.R., Warner G.G. (1969). Estimates of absorbed fractions for monoenergetic photon sources uniformly distributed in various organs of a heterogeneous phantom. J. Nucl. Med..

[11] Han M.C., Kim J., Hong C.S., Chang K.H., Han S.C., Park K., Kim D.W., Kim H., Chang J.S., Kim J. (2022). Performance evaluation of deformable image registration algorithms using computed tomography of multiple lung metastases. Technol. Cancer Res. Treat..

[12] Al-Zein A., Naim R.H., Jalbout W., Shahine B.H. (2025). Performance evaluation and quantitative comparison of two 4DCT imaging respiratory systems using deformable image registration. J. Appl. Clin. Med. Phys..

[13] Zeng H., Xiangyu E., Lv M., Zeng S., Feng Y., Shen W., Guan W., Zhang Y., Zhao R., Yu J. (2025). Deep learning-based synthetic CT for dosimetric monitoring of combined conventional radiotherapy and lattice boost in large lung tumors. Radiat. Oncol..

[14] Chamunyonga C., Burbery J., Caldwell P., Rutledge P., Fielding A., Crowe S. (2018). Utilising the Virtual Environment for Radiotherapy Training System to Support Undergraduate Teaching of IMRT, VMAT, DCAT Treatment Planning, and QA Concepts. J. Med. Imaging Radiat. Sci..

[15] Hindley N., Keall P.J. (2025). An open-source deep learning framework for respiratory motion monitoring and volumetric imaging during radiation therapy. Med. Phys..

[16] Huang Y., Dong Z., Wu H., Li C., Liu H., Zhang Y. (2022). Deep learning-based synthetization of real-time in-treatment 4D images using surface motion and pretreatment images: A proof-of-concept study. Med. Phys..

[17] Bolch W., Lee C., Wayson M., Johnson P. (2010). Hybrid computational phantoms for medical dose reconstruction. Radiat. Environ. Biophys..

[18] Ahmad R., Cantwell J., Borrelli C., Lim P., D’Souza D., Gaze M.N., Moinuddin S., Gains J., Veiga C. (2024). Development of age-specific population-based paediatric computational phantoms for image-based data mining and other radiotherapy applications. Biomed. Phys. Eng. Express.

[19] Lee C., Lee C., Park S. (2006). –H.; Lee, J.–K. Development of the two Korean adult tomographic computational phantoms for organ dosimetry. Med. Phys..

[20] Chu P.W., Kofler C., Mahendra M., Wang Y., Chu C.A., Stewart C., Delman B.N., Haas B., Lee C., Bolch W.E. (2023). Dose length product to effective dose coefficients in children. Pediatr. Radiol..

[21] Alem-Bezoubiri A., Bezoubiri F., Speiser M., Suleiman S.A., Donya H., Chami A.C. (2025). Monte Carlo study of organ doses and related secondary cancer risk estimations for patients undergoing prostate radiotherapy: Algerian population-based study. Appl. Radiat. Isot..

[22] Owens C.A., Rigaud B., Ludmir E.B., Gupta A.C., Shrestha S., Paulino A.C., Smith S.A., Peterson C.B., Kry S.F., Lee C. (2022). Development and validation of a population-based anatomical colorectal model for radiation dosimetry in late effects studies of survivors of childhood cancer. Radiother. Oncol..

[23] Somerwil P.C., Nout R.A., Mens J.M., Kolkman-Deurloo I.K., van Beekhuizen H.J., Dankelman J., van de Berg N.J. (2021). An anthropomorphic deformable phantom of the vaginal wall and cavity. Biomed. Phys. Eng. Express.

[24] Jiang S., Yang S., Yang Z., Zhang D., Luan Y., Zhou Z. (2025). A Personalized Tumor kV-IORT Navigation System Based on Hybrid Twin. J. Appl. Clin. Med. Phys..

[25] (2001). ICRU Report 64: Dosimetry of High-Energy Photon Beams based on Standards of Absorbed Dose to Water. https://journals.sagepub.com/toc/crua/1/1.

[26] Shariff M., Grigo J., Masitho S., Brandt T., Weiß A., Lambrecht U., Stillkrieg W., Lotter M., Putz F., Fietkau R. (2024). End to end testing for stereotactic radiotherapy including the development of a multimodality phantom. Z. Med. Phys..

[27] Kodama S., Iijima K., Okamoto H., Nishioka S., Sakasai T., Igaki H., Itami J. (2021). Performance of a newly designed end to end phantom compatible with magnetic resonance guided radiotherapy systems. Med. Phys..

[28] Singhrao K., Fu J., Wu H.H., Hu P., Kishan A.U., Chin R.K., Lewis J.H. (2020). A novel anthropomorphic multimodality phantom for MRI-based radiotherapy quality assurance testing. Med. Phys..

[29] Steinmann A., Alvarez P., Lee H., Court L., Stafford R., Sawakuchi G., Wen Z., Fuller C.D., Followill D. (2020). MRIgRT head and neck anthropomorphic QA phantom: Design, development, reproducibility, and feasibility study. Med. Phys..

[30] Sun J., Dowling J., Pichler P., Menk F., Rivest-Henault D., Lambert J., Parker J., Arm J., Best L., Martin J. (2015). MRI simulation: End-to-end testing for prostate radiation therapy using geometric pelvic MRI phantoms. Phys. Med. Biol..

[31] Mei Y., Lakotsenina E., Wegner M., Hehne T., Krause D., Hakimeh D., Wu D., Schültke E., Hausmann F., Kurreck J. (2024). Three-Dimensional-Bioprinted Non-Small Cell Lung Cancer Models in a Mouse Phantom for Radiotherapy Research. Int. J. Mol. Sci..

[32] Mei Y., Wu D., Berg J., Tolksdorf B., Roehrs V., Kurreck A., Hiller T., Kurreck J. (2023). Generation of a perfusable 3D lung cancer model by digital light processing. Int. J. Mol. Sci..

[33] The Alderson Radiation Therapy Phantom—Radiology Support Devices Inc. https://rsdphantoms.com/product/the-alderson-radiation-therapy-phantom/.

[34] Tillery H., Moore M., Gallagher K.J., Taddei P.J., Leuro E., Argento D., Moffitt G., Kranz M., Carey M., Heymsfield S.B. (2022). Personalized 3D-printed anthropomorphic whole-body phantom irradiated by protons, photons, and neutrons. Biomed. Phys. Eng. Express..

[35] Tanabe Y., Iseri T., Onizuka R., Ishida T., Eto H., Nakaichi M. (2023). Improving animal-specific radiotherapy quality assurance for kilovoltage X-ray radiotherapy using a 3D printed dog skull water phantom. Open Vet. J..

[36] Bajrami D., Spano F., Wei K., Bonmarin M., Rossi R.M. (2025). Human skin models in biophotonics: Materials, methods, and applications. Adv. Healthc. Mater..

[37] Pogue B.W., Patterson M.S. (2006). Review of tissue simulating phantoms for optical spectroscopy, imaging and dosimetry. Biomed. Opt..

[38] Bustillo J.P.O., Mata J.L., Posadas J.R.D., Inocencio E.T., Rosenfeld A.B., Lerch M.L.F. (2025). Characterization evaluation methods of fused deposition modeling and stereolithography additive manufacturing for clinical linear accelerator photon and electron radiotherapy applications. Phys. Med..

[39] Ahmed A.M.M., Buschmann M., Breyer L., Kuntner C., Homolka P. (2024). Tailoring the mass density of 3D printing materials for accurate X-ray imaging simulation by controlled underfilling for radiographic phantoms. Polymers.

[40] McGarry C.K., Grattan L.J., Ivory A.M., Leek F., Liney G.P., Liu Y., Miloro P., Rai R., Robinson A.P., Shih A.J. (2020). Tissue mimicking materials for imaging and therapy phantoms: A review. Phys. Med. Biol..

[41] Tino R., Yeo A., Leary M., Brandt M., Kron T. (2019). A systematic review on 3D-printed imaging and dosimetry phantoms in radiation therapy. Technol. Cancer Res. Treat..

[42] Kamomae T., Shimizu H., Nakaya T., Okudaira K., Aoyama T., Oguchi H., Komori M., Kawamura M., Ohtakara K., Monzen H. (2017). Three-dimensional printer-generated patient-specific phantom for artificial in vivo dosimetry in radiotherapy quality assurance. Phys. Med..

[43] Frerker B., Engels E., Paino J., Rover V., Bustillo J.P., Wegner M., Cameron M., Fiedler S., Häusermann D., Hildebrandt G. (2025). Fast and fractionated: Correlation of dose attenuation and the response of human cancer cells in a new anthropomorphic brain phantom. Biomimetics.

[44] Breslin T., Paino J., Wegner M., Engels E., Fiedler S., Forrester H., Rennau H., Bustillo J., Cameron M., Häusermann D. (2023). A novel anthropomorphic phantom composed of tissue-equivalent materials for use in experimental radiotherapy: Design, dosimetry and biological pilot study. Biomimetics.

[45] Wegner M., Frenzel T., Krause D., Gargioni E. (2023). Development and characterization of modular mouse phantoms for end-to-end testing and training in radiobiology experiments. Phys. Med. Biol..

[46] Tino R.B., Yeo A.U., Brandt M., Leary M., Kron T. (2022). A customizable anthropomorphic phantom for dosimetric verification of 3D-printed lung, tissue, and bone density materials. Med. Phys..

[47] ICRU Tissues. https://www.qrm.de/en/products/icru-tissues.

[48] Ma X., Buschmann M., Unger E., Homolka P. (2021). Classification of X-Ray Attenuation Properties of Additive Manufacturing and 3D Printing Materials Using Computed Tomography From 70 to 140 kVp. Front. Bioeng. Biotechnol..

[49] Mei K., Pasyar P., Geagan M., Liu L.P., Shapira N., Gang G.J., Stayman J.W., Noël P.B. (2023). Design and fabrication of 3D-printed patient-specific soft tissue and bone phantoms for CT imaging. Sci. Rep..

[50] https://www.ptwdosimetry.com/en/products/microdiamond.

[51] Petasecca M., Cullen A., Fuduli I., Espinoza A., Porumb C., Stanton C., Aldosari A.H., Brauer-Krisch E., Requardt H., Bravin A. (2012). X-Tream: A novel dosimetry system for Synchrotron Microbeam Radiation Therapy. J. Instrum..

[52] Carlsson Tedgren A., Carlsson G.A. (2009). Influence of phantom material and dimensions on experimental 192Ir dosimetry. Med. Phys..

[53] Li X., Gou J., Santhanam A.P., Maiti C., Ilegbusi O.J. (2025). Tissue mimicking hydrogel foam materials with mechanical and radiological properties equivalent to human lung. Sci. Rep..

[54] Jiang S., Liu S., Feng W. (2011). PVA hydrogel properties for biomedical application. J. Mech. Behav. Biomed. Mater..

[55] Malone A.J., Cournane S., Naydenova I.G., Fagan A.J., Browne J.E. (2020). Polyvinyl alcohol cryogel based vessel mimicking material for modelling the progression of atherosclerosis. Phys. Med..

[56] Vanina A.S., Lavrova A.I., Safonov D.A., Sychev A.V., Proskurkin I.S., Postnikov E.B. (2024). Mimicking marker spread after disruption of the blood–brain barrier with a collagen-based hydrogel phantom. Biomimetics.

[57] Baker A.J.A., Galindo E.J., Angelos J.D., Salazar D.K., Sterritt S.M., Willis A.M., Tartis M.S. (2023). Mechanical characterization data of polyacrylamide hydrogel formulations and 3D printed PLA for application in human head phantoms. Data Brief.

[58] Sunnuclear Dynamic Thorax Brochure. https://www.sunnuclear.com/uploads/documents/datasheets/Dynamic-Thorax-Brochure_103024.pdf.

[59] World Health Organization Lung Cancer—Fact Sheet. https://www.who.int/news-room/fact-sheets/detail/lung-cancer.

[60] Wheatley M., de Deene Y. (2022). A novel anthropomorphic breathing phantom with a pneumatic MR-safe actuator for tissue deformation studies during MRI and radiotherapy. Phys. Medica.

[61] Kim J., Lee Y., Shin H., Ji S., Park S., Kim J., Jang H., Kang Y. (2016). Development of deformable moving lung phantom to simulate respiratory motion in radiotherapy. Med. Dosim..

[62] Colvill E., Krieger M., Bosshard P., Steinacher P., Rohrer Schnidrig B.A., Parkel T., Stergiou I., Zhang Y., Peroni M., Safai S. (2020). Anthropomorphic phantom for deformable lung and liver CT and MR imaging for radiotherapy. Phys. Med. Biol..

[63] Mawatari S., Oku Y., Toyota M. (2025). Comparison of target phase positioning with respiratory motion between four-dimensional CT and four-dimensional cone beam CT: A phantom study. Nihon Hoshasen Gijutsu Gakkai Zasshi.

[64] Sahin S., Ozen S.K., Ertan F., Sahiner E. (2025). Design and manufacturing of a dynamically deformable liver phantom for radiotherapy. Appl. Radiat. Isot..

[65] Malin E.J., Ceritoglu C., Abadi E., Ratnanather J.T., Samei E., Segars W.P. (2025). Library of realistic 4D digital beating heart models based on patient CT data. Med. Phys..

[66] Gregg K.W., Ruff C., Koenig G., Penev K.I., Shepard A., Kreissler G., Amatuzio M., Owens C., Nagpal P., Glide-Hurst C.K. (2024). Development and first implementation of a novel multi-modality cardiac motion and dosimetry phantom for radiotherapy applications. Med. Phys..

[67] Ali I.G., El Naqa I. (2025). The Biophysics of Flash Radiotherapy: Tools for Measuring Tumor and Normal Tissues Microenvironment. Antioxidants.

[68] Leung J.K., Panchal R., Anbalagan S., Durie E.P., Mansfeld J., Somaiah N. (2025). Modulating Redox Biology to Improve Radiation Responses. Cancer J..

[69] Wu Q., Ren W., Yu Z., Dong E., Zhang S., Xu R.X. (2015). Microfabrication of polydimethylsiloxane phantoms to simulate tumor hypoxia and vascular anomaly. Biomed. Opt..

[70] Liu L., Wu D., Qian Z., Jiang Y., You Y., Wang Y., Zhang F., Ning X., Mei J., Iqbal J. (2025). Empowering hypoxia to convert cold tumors into hot tumors for breast cancer immunotherapy. Cell Death Discov..

[71] Pignatta S., Cortesi M., Arienti C., Zanoni M., Cocchi C., Sarnelli A., Arpa D., Piccinini F., Tesei A. (2020). Effects of radiotherapy and short-term starvation combination on metastatic and non-tumor cell lines. DNA Repair.

[72] McHugh D.J., Zhou F.L., Wimpenny I., Poologasundarampillai G., Naish J.H., Hubbard Cristinacce P.L., Parker G.J.M. (2018). A biomimetic tumor tissue phantom for validating diffusion-weighted MRI measurements. Magn. Reson. Med..

[73] Wang Y., Zhang F., Qian Z., Jiang Y., Wu D., Liu L., Ning X., Mei J., Chen D., Zhang Y. (2025). Targeting collagen to optimize cancer immunotherapy. Exp. Hematol. Oncol..

[74] Chavez L., Gao S., Pandey V., Yuan N., Ragab S., Li J., Hepburn M.S., Smith P., Edelheit C., Corr D.T. (2025). Design and characterization of an optical phantom for mesoscopic multimodal fluorescence lifetime imaging and optical coherence elastography. Biomed. Opt. Express.

[75] Cho G.Y., Kim S., Jensen J.H., Storey P., Sodickson D.K., Sigmund E.E. (2012). A versatile flow phantom for intravoxel incoherent motion MRI. Magn. Reson. Med..

[76] Yu H.B., Han B.J., Hu J.Q., Luo Y., Liu H.Y., Zhang X.Y., Li Y., Liu R., Hua B.J. (2025). Worldwide research on 3D printing for cancer: A dual-method analysis of bibliometrics and stratified focused thematic. Int. J. Surg..

[77] Bustillo J.P.O., de Rover V., Engels E.E.M., Cayley J., Cameron M., Long S., Carolan M., Frerker B., Lerch M.L.F., Schültke E. (2025). A canine head phantom for quality assurance in multiport microbeam radiation therapy: Animal phantom fabrication and dosimetry protocol. Biomed. Phys. Eng. Express.

[78] Constantinou C., Attix F.H., Paliwal B.R. (1982). A solid water phantom material for radiotherapy X-ray and gamma-ray beam calibrations. Med. Phys..

[79] Ramaseshan R., Heydarian M. (2008). Dosimetric evaluation of Plastic Water diagnostic therapy. J. Appl. Clin. Med. Phys..

[80] Gargett M.A., Briggs A.R., Booth J.T. (2020). Water equivalence of a solid phantom material for radiation dosimetry applications. Phys. Imaging Radiat. Oncol..

[81] Manchado de Sola F., Vilches M., Prezado Y., Lallena A.M. (2018). Impact of cardiosynchronous brain pulsations on Monte Carlo calculated doses for synchrotron micro- and minibeam radiation therapy. Med. Phys..

[82] Liu H.H., Koch N., Starkschall G., Jacobson M., Forster K., Liao Z., Komaki R., Stevens C.W. (2004). Evaluation of internal lung motion for respiratory-gated radiotherapy using MRI: Part II-margin reduction of internal target volume. Int. J. Radiat. Oncol. Biol. Phys..

[83] Davidson S.E., Ibbott G.S., Prado K.L., Dong L., Liao Z., Followill D.S. (2007). Accuracy of two heterogeneity dose calculation algorithms for IMRT in treatment plans designed using an anthropomorphic thorax phantom. Med. Phys..

[84] Yadav N., Singh M., Mishra S.P., Ansari S. (2023). Development of an Anthropomorphic Heterogeneous Female Pelvic Phantom and Its Comparison with a Homogeneous Phantom in Advance Radiation Therapy: Dosimetry Analysis. Med. Sci..

[85] Fogliata A., Cozzi L. (2017). Dose calculation algorithm accuracy for small fields in non-homogeneous media: The lung SBRT case. Phys. Med..

[86] Monti di Sopra F., Keall P., Beckham W. (1998). Comparing dose calculation algorithms for an orthovoltage beam in a bone phantom. Australas. Phys. Eng. Sci. Med..

[87] Hoffmann L., Linaa M.B., Møller D.S. (2025). Independent secondary dose calculation for patient-specific quality assurance: Quantitative benefit of Monte-Carlo and custom beam modeling. J. Appl. Clin. Med. Phys..

[88] Mentzel F., Kröninger K., Lerch M., Nackenhorst O., Rosenfeld A., Tsoi A.C., Weingarten J., Hagenbuchner M., Guatelli S. (2022). Small beams, fast predictions: A comparison of machine learning dose prediction models for proton minibeam therapy. Med. Phys..

